# An In Vitro Comparison of Different Diagnostic Methods in Detection of Residual Dentinal Caries

**DOI:** 10.1155/2010/864935

**Published:** 2010-06-03

**Authors:** Nimet Unlu, Rabia Banu Ermis, Sevgi Sener, Ebru Kucukyilmaz, Ali Riza Cetin

**Affiliations:** ^1^Department of Conservative Dentistry, Faculty of Dentistry, Selcuk University, Konya 42075, Turkey; ^2^Department of Conservative Dentistry, Faculty of Dentistry, Suleyman Demirel University, Isparta 32060, Turkey; ^3^Department of Radiology and Oral Diagnosis, Faculty of Dentistry, Selcuk University, Konya 42075, Turkey; ^4^Department of Pedodontics, Faculty of Dentistry, Selcuk University, Konya 42075, Turkey

## Abstract

The aim of this study was to investigate the efficiency of different diagnostic methods in detection of residual dentinal caries in excavated cavities. Fifty extracted molar with deep dentinal carious lesions were excavated using a slow-speed handpiece. All cavities were assessed by laser fluorescence(LF) device, electronic caries monitor(ECM), and caries detector dye(CDD) by three independent observers blindly. The measurements were repeated after two weeks. Specimens containing dentin slices 150 *μ*m in thickness were prepared for histological analyses. The existence and absence of carious dentin was determined using a lightmicroscope. The average intraobserver accuracy was 1.00 (perfect agreement) for CDD, 0.86 (excellent agreement) for ECM, and 0.50 (good agreement) for LF. The average interobserver accuracy values were 0.92 (excellent agreement), (0.36 marginal agreement) and 0.48 (good agreement), for CDD, ECM, and LF, respectively. The average specificity was 0.60 for CDD, 73% for ECM, and 0.50 for LF. The average sensitivity was 0.55 for CDD, 0.85 for LF, and 0.47 for ECM. The average accuracy values were 0.53, 0.51, and 0.81 for CDD, ECM, and LF, respectively. LF had the greatest sensitivity and accuracy values of any of the methods tested. As a conclusion, LF device is appeared to most reliable method in detection of remain caries in cavity. However, because of its technical sensitivity it may susceptible to variations in measurements. To pay attention to the rule of usage and repeated measurements can minimize such variations in clinical practice. It was concluded that LF is an improvement on the currently available aids for residual caries detection.

## 1. Introduction

The main aim of a dentist is to remove the irreversibly demineralised superficial tissue and to remove the highly infected biomass of carious dentin during the carious process. New restorative materials are difficult to adhesion on this heavily infected dentin tissue, if it is retained at the cavity floor. Thus, long-term prognosis of the final restoration is adversely affected [1, 2]. This assumes that the bacteria within the dentin are important. However, the weight of experimental evidence might suggest that the bacteria in the biofilm are what matters [3], although some authors accept that this statement is controversial. It appears that lesion progression can be arrested either by simple removal of the biofilm or by the sealing of bacteria within the cavity and restoration of the tooth so that regular removal of the biofilm is possible [2]. The discussion as to how much tissue must be removed in order to arrest the caries process is not new. In 1859, Tomes [4] wrote “it is better that a layer of discolored dentine should be allowed to remain for the protection of the pulp rather than run the risk of sacrificing the tooth”, but in 1908, Black [5] disagreed claiming “... it is better that it is removed of all decayed dentine overlaying”.

Currently, physical criterion, used most commonly by dental practitioners to guide clinical excavation of this infected, demineralised that dentin is hardness/texture of the tissue, although some dentists may take into account its color and may use caries detector dyes [6–8]. All of these criteria suffer from the subjectivity inherent between dentists in clinical practice, which is likely to result in variations in the quality and quantity of dentin removed during operative intervention. However, at the chairside they have limited clues which include the subjective criteria of color and relative hardness of the carious dentin [9]. Therefore, the reproducibility of these evaluations between examiners is low [10]. In addition, when caries detection dyes, which generally consist of 1% acid red in propylene glycol [9, 11], are used in clinical situations, the dentin that becomes stained light pink should not be removed to prevent excessive dentin removal [11]. However, the degree of light-pink dentin staining in deep carious layers is difficult to evaluate objectively by visual inspection alone. Consequently, when removing demineralised dentin, it is not always easy to know at what point to stop excavation because there is an apparent lack of objective clinical markers. These variations may have clinical implication including differences in the size of the cavities and produced the pulpal health beneath prepared cavities and the strength of the remaining tooth structure. It seems sensible, therefore, to develop an objective marker for excavationable carious dentin [1]. Therefore, to ensure minimal tooth substance removal during clinical restorative treatment based on the concept of minimal intervention dentistry, an important need to assess the effect of some new objective caries detection techniques to aid in differentiating heavily infected dentin from affected dentin during excavation. 

In recent years, new techniques such as electrical conductivity measurement (ECM) and laser fluorescence (LF) devices have been developed for objective caries detection [12–14]. The principle of the use of LF is based upon the principle of the laser fluorescence emitted from carious surfaces which is greater than that emitted from sound surfaces [15]. The ECM device measures the bulk resistance of tooth tissue. As the tooth demineralised in caries process, the loss of mineral leads to increased porosity in the tooth structure. Increased porosity leads to decreased electrical resistance [16]. Although the performance and reproducibility of these devices have been investigated on occlusal and proximal surfaces in vitro [17], and in vivo [18], there was limited information about the validity of LF [19, 20], and no information about the validity of ECM in detection of the residual caries after the excavation. Furthermore, no previous studies have reported the comparison of the sensitivity, specificity, and intra- and interobserver agreement of ECM, LF, and CDD in detecting of residual caries.

To compare these different diagnostic methods is also important for the objective identification of residual caries after carious dentin removal with conventional bur excavation. The main aim of this study was to compare histologically the efficiency of ECM, LF, and CDD in detection of residual dentinal caries in excavated cavities. Also, other aim was to assess the accuracy, sensitivity, specificity, and inter- and intraobserver accuracy of these diagnostic methods in detecting of residual caries in cavity preparation.

## 2. Methods and Materials

### 2.1. Specimens

The protocol of this investigation was approved by the Ethics Committee of the Faculty of Dentistry, Selcuk University (Process No. 61/2009). Several private dental offices provided fifty freshly extracted human molar teeth with coronal dentin that include active caries and without developmental defects, restoration, plaque, cracking, and discoloration were used in this study. In each office, the teeth were extracted for periodontal, orthodontic, or prosthodontic reasons and were obtained from patients who consented to their use for research. Teeth having radiographically D3, caries limited to the outer half of the dentin were selected [21]. Immediately after extraction teeth were stored in saline solution without any antibacterial substance at 8°C [22–24] until further processing. Before using, all soft periodontal tissue and extrinsic deposits on teeth were removed using a hand scaler, and teeth were cleaned with pumice slurry.

### 2.2. Caries Removal

Enamel were removed by grinding the sample teeth perpendicularly to the carious surface using a slow-speed diamond saw under continuous water cooling until a flat surface was formed and the lesion in dentin was exposed. After that, caries dentin was mechanically removed with conventional round steel burs (ISO #012; ELA, Engelskirchen, Germany) in a contra-angle speed- reducing hand piece (400 rpm). The hardness of dentine was checked with a dental explorer (Jensen, Germany). This was repeated until either a leathery-hard texture was reached or a sharp scratching sound was heard in all teeth when each checked with a dental explorer [20, 25]. The process of caries removal in all groups has been carried out by the same examiner (A.R.C.). Then, the cavity inspection for the successful removal of caries was carried out by three independent observers by direct visual examination using the criteria of Kidd et al. [7]. 

**Figure 1:**
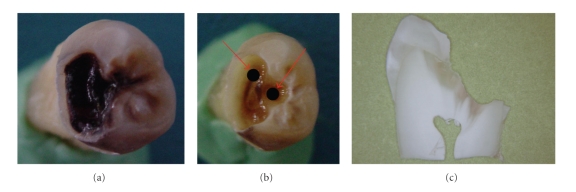
(a) D3 caries: (b) After caries dentin was mechanically removed, to ensure that the same spots were analyzed each time, two signs were done on photographs with a black pen (c) histological section.

**Figure 2:**
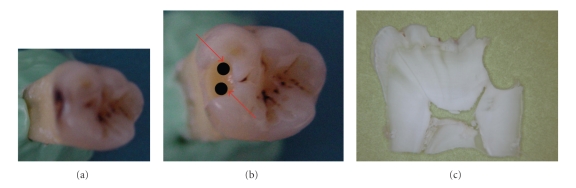
(a) D3 caries: (b) After caries dentin was mechanically removed, to ensure that the same spots were analyzed each time, two signs were done on photographs with a black pen (c) histological section.

After completion of caries removal, digital images of the teeth were taken from the surfaces under investigation with a digital camera (Figures 1(a), 1(b), 1(c) and Figures 2(a), 2(b), 2(c)). These images were stored on a computer; the area to be evaluated was indicated on photographs with two black dots by using a pen to identify the location for the second measurements and to ensure that the same spots were analyzed each time [20] (Figures 1(b) and 2(b)). Before each measurement, photographs with black dot of each tooth were seen to ensure that the same spots were analyzed each time.

### 2.3. Examinations

All cavities were assessed by using laser fluorescence device (LF, DIAGNOdent pen, KaVo, Biberach, Germany), electronic caries monitor (ECM IV; Lode Diagnostics, Glaningen, The Netherlands), caries detector dye (CDD; Quadrant CariTest, Cavex, USA), and histological examination by three independent observers blindly. The examinations were carried out as follows: 

#### 2.3.1. LF Examination

LFpen (DIAGNOdent pen, KaVo, Biberach, Germany) was used for the laser fluorescence measurements. Probe was used for smooth surfaces. Each tooth was retrieved from the saline solution, wiped with a paper tissue, dried in air for approximately 10 seconds, and then measured by LFpen accordance with the manufacturer's instructions. Tip was placed on the site and rotated around its vertical axis. LFpen device was calibrated against the porcelain reference object before each measurement session. The fluorescence of a sound spot on smooth surface of the tooth was measured in order to provide a baseline reading for each tooth (secondary calibration) and again after every 10th tooth. LF values were carefully measured with the apex of the tip in contact with the surface of the carious dentin of the cavity floor of the approximal or occlusal cavities. The samples were dried briefly using compressed air. The highest LF readings from the marked two lesion area were recorded for each sample. The sites were indicated and controlled as dots on the photograph. Three blinded observers evaluated LF readings within the same groups. Two measurements in each surface were taken and the mean value was calculated. Some studies reported that the LF cut-off point for dentin caries was from 18 to 30 [14, 20, 26, 27]. In this study, the LF cut-off point was chosen according to similar these studies. It was already stated that the borderline was set at value of about 30 for operative intervention [27]. The cut-off points were determined as follows [14]; 0 to 29 = no caries, 30 and over = dentinal caries (residual caries).

The status of the two marked areas on proximal or occlusal sites on each tooth was assessed by three observers. Three measurements were done in each surface site and the mean value was calculated. After an interval of 2 weeks, same procedure was repeated by the same operator, under identical conditions, without access to the data from the previous session.

#### 2.3.2. ECM Examination

For measurements with ECM (ECM IV; Lode Diagnostics, Glaningen, The Netherlands) both, the tooth and reference electrode were held in the same hand without direct contact with each other. A measurement was made in accordance with the manufacturer's instructions by touching the approximal or the occlusal site indicated on the black dots with the instrument probe. The air-flow was 5 L/min during 5 seconds. Data shown on the front panel of the instrument were registered.

#### 2.3.3. Dye Examination

After visual, LF, and ECM examinations, the presence or absence of residual caries was also detected using a CDD. Infected demineralized dentin will show definite dye stains, whereas noninfected dentin will not take up much of the dye [28, 29]. The dye was applied to the approximal or occlusal cavities using a small brush and removed after 10 seconds by a 5-second water spray. The cavities were then examined under a Light microscope (×2 magnification, Olympus BX 50, Japan) for any dentin site stained by the dye. All observers were calibrated to the color of stained dentin on marked sites on each tooth as follow as 0 = white (sound) and 1 = blue (carious).

#### 2.3.4. Histological Examination

After all the examinations had been completed, color photographs of the caries removal surfaces were taken to assist the subsequent histological examination. Prior to the histological examination, the specimens' roots were separated from the coronal part and each tooth was prepared for histological examination. Teeth were hemisectioned in a mesial-distal direction through the center of the marked lesion area with a high-speed drill and fine diamond bur. Speed was set at 800 rpm and a moderate weight (100 g) was chosen to guide the diamond-coated saw blade during the cutting procedure. One half of each tooth was processed for histological evaluations (Figures 1 and 2). Subsequently, specimens were dried in alcohol solvents of increasing concentration and embedded in Technovit 9100 New (Heraeus Kulzer, Wehrheim, Germany). Serial sections of 200 *μ*m were prepared and polished to a final thickness of approximately 150 *μ*m. The specimens were immersed in water and wet sections were viewed under a polarized light microscope (Axiovert 135, Carl Zeiss, Jena, Germany) at ×10 magnification for the presence or absence of residual caries. The existence or absence of residual caries was determined by two observers blindly. To prevent observer bias, the histologic validation was carried out by two observers one month after the diagnostic assessment. In cases of disagreement, a third observer assessed the test sites. The status of demineralized zone was then determined by majority opinion [20, 30].

### 2.4. Statistical Analysis

Specificities and sensitivities were calculated for each diagnostic method using the histologic gold standard. Intra- and interobserver accuracy values were assessed using Kappa statistics [31, 32]. The classification of Kappa values was performed using criteria proposed in previous report [35, 36]: Kappa value 1 = perfect agreement, Kappa values above 0.75 = excellent agreement, Kappa values from 0.4 to 0.75 = good agreement and Kappa values below 0.4 = marginal agreement.

Accuracy was calculated by the following formula in cross-tabs: the number of true positive values + the number of true negative values/total number.

## 3. Results

The histological examination of 50 teeth (100 test areas) revealed that 12 (12%) test sites had residual caries. For three observers, Table 1 presents the average sensitivity and specificity values of all diagnostic methods. Although the LF had the highest sensitivity value (0.85), it had the lowest specificity value (0.50). The lowest sensitivity (0.47) and the highest specificity (0.74) values were observed in ECM. The specificity and sensitivity values of CDD were similar (0.55 and 0.60, resp.).

Table 2 shows the intra- and interobserver accuracy values of three observers for all diagnostic methods. The highest intra- and interobserver accuracy values were observed in CDD. The average intra- and interobserver accuracy values of CDD were 1.00 (perfect agreement) and 0.92 (excellent agreement), respectively. The average intra- and interaccuracy values of LF were 0.50 (good agreement), and 0.48 (good agreement) respectively. The lowest average interobserver accuracy value (0.36, marginal agreement) was observed in ECM. However, the average intraobserver accuracy value of ECM was 0.86 (excellent agreement). 

The average accuracy value was 0.53 (min = 0.52, max = 0.56) for CDD, 0.51 (min = 0.36 max = 0.62) for ECM, and 0.81 (min = 0.76, max = 0.86) for LF. LF had the highest accuracy in detecting residual caries in this study. 

There was no significant correlation between the gold standard and CDD (*P* > .05  for all observers) and between the gold standard and ECM (*P* > .05 for observer 1, *P* > .05 for observers 2 and 3). Therefore, there was a significant difference between the measurement of gold standard and CDD or ECM. There was found significant correlation between the gold standard and LF (*P* = .001 for all observers).

## 4. Discussion

During caries removal in a clinical situation, visual inspection of the color of carious tissue, a caries detector dye, and detection with an excavator are used. Whereas a significant correlation has been found between dentin hardness and level of bacterial infection, the same is not true for color [7]. However, determining both tissue color and hardness is highly subjective. Carious regions can easily be overlooked and deciding whether excavation is complete or not is often difficult. Therefore, an objective and accurate technique to aid the clinician in differentiating heavily infected dentin from affected dentin during excavation is still needed. Consequently, this present study aimed to investigate the efficiency of different diagnostic methods in detection of residual dentinal caries in excavated cavities.

The objective identification of this infected/affective dentin carious boundary is also important for the dental researcher who wishes to determine the remaining dentin carious after caries excavation. Some authors maintained that the simple histological analysis of a hemisected, carious lesion is difficult to interpret and it is not possible to distinguish the histological boundary in question [1, 2]. However, using histopathological and microhardness evaluation, Mendes et al. [21] showed that the best LF performance was obtained at D_3, _caries limited to the outer half of the dentin threshold. Further, Yazici et al. [20] stated that LF agreed better than the caries detector dye with the histological evaluation in assessing teeth residual caries. However, using histological and scanning electron microscope analyses, Gurbuz et al. [30] stated that LF abilities to detect the residual caries were low after hand excavation and chemomechanical caries removal, and therefore, it is advisable to test the residual caries with an additional diagnostic tool such as visual tactile examination. Thus, in the present study, we aimed to compare histologically the effectiveness of different diagnostic methods, such as ECM, LF, and CDD, on identification of residual caries. Contrarily, some studies documenting the diagnosis of caries using the LF [14, 19, 35] reported that histological evaluation as a gold standard is subjective, for example, in regard to enamel and dentin caries. Iwami et al. [23], using the rates of caries detection as a gold standard clearly, have showed the relationship between LF values of ground dentin surfaces and the rates of bacterial detection. Also, they reported that there are some limitations in using LF for evaluating caries removal. The results of this present study showed that LF is advisable to test the residual caries but it should be improved to prevent the overexcavation.

Previous studies on LF [12, 13, 36, 37] have established some cut-off points for LF in the diagnosis of dentin caries, and the manufacturer also states cut-off points in their literature on the clinical use of LF, but these cut-off points were different from each other. According to a clinical study of Lussi et al. [14] the cut-off values advised for clinical use of LF in dentin caries were ≥30 for operative intervention. They suggested that the border-line reading for operative intervention reduces the sensitivity of the device but increases its specificity and that a higher setting of this trigger for operative intervention also represents a safety for initially carious cases. However, the results of Iwami et al.'s in vitro study showed that no bacteria were detected at LF cut-off value of less than 15.6 [23]. In their study, this value was considered a cut-off point for the complete removal of carious dentin. Also, they stated that this cut-off value was obtained from the results of a limited number of specimens and cannot be generalized [23]. However, Kidd and Fejerskov [2] reviewed that there is little evidence related with infected dentine that must be removed prior to sealing the tooth. In light of these studies, we determined ≥30 as of the cut-off points of LF in detection of residual dentin carious in order to prevent overexcavation. 

Caries detecting dyes have been used to differentiate clinically “infected” from “affected” dentine during caries removal [10]. The use of these dyes, however, does not provide a completely objective method for assessment of caries removal. Pitts [10] reported that the more superficial zone of infected dentine was an irreversibly damaged, bacterially infected layer that would never remineralize. The deepest affected dentine was shown to harden as a result of remineralization [38]. Fusayama's group suggested the dye staining front coincided with the bacterial invasion of the dentine. However, several studies have reported that the dye does not discretely discriminate the bacterially infected from softened affected tissues [6, 39, 40]. Also, Banerjee et al. [41] reported that the use of dyes is not routinely advocated in lesions extending into the middle third of dentin or deeper due to the increased risk of unnecessary and often avoidable pulpal involvement during cavity preparation. Consequently, its injudicious use may lead to overpreparation of the tissues, encouraging excess removal at the enamel-dentine junction as well as unnecessary removal of dentine over the pulpal surface [42]. Previous studies [19, 20], which were assessment of residual caries in excavated cavity, reported that the sensitivity values were 0.40 and 0.65, and the specificity values were 0.1 and 0.17 for CCD. In this present study, intra- and interobserver agreements were significantly higher than the other methods but average sensitivity value was 0.55 and specificity value was 0.60 for CDD. The average specificity value of CDD was similar with LF, but average sensitivity value of CDD was the lowest than LF. CDD has the lowest accuracy value in this study. Consistently with other studies, the results of this present study showed that CDD should be used with caution to test the residual caries in D_3_ level dentin carious and the use of CDD can result in overexcavation or incomplete removal of the carious lesion. 

Therefore, restoration applying minimal intervention dentistry is needed for the least amount of enamel and dentin having to be removed. Recently, new diagnostic devices, such as the LF and ECM, were developed for objective detection of caries before removal and have been reported as useful devices in objective evaluation of occlusal caries in vitro. The new diagnostic methods have concentrated mostly on detecting occlusal and hidden caries. Lussi et al. [14] have reported that carious dentin may be evaluated by the DIAGNOdent during removal of caries as well as by caries detector dyes. However, in the literature, there are few studies which measured the reliability and validity of laser fluorescence in assessment of residual caries after caries removal [19, 20, 30]. It has been observed that LF does not adequately measure small mineral changes [12, 13]. Recently, several studies reported that the LF readings reflect changes in the organic matrix rather than in the inorganic content of the teeth [12, 13, 21]. They found that the best LF performance was obtained at the D3 threshold. It has been shown that the LF readings were useful to facilitate accurate removal of the caries-infected dentin [26]. Also, in present study, was compared the effectiveness of different diagnostic methods to identification of residual caries in D_3  _level carious lesions.

ECM has been proposed for caries lesion detection and its measurement depends on the permeability changes due to demineralization of the tissues. In in vitro and in vivo studies the diagnostic performance of ECM has been evaluated [43] but no information is available about the accuracy of ECM in assessment of residual caries in literature. In this study, we aimed also to investigate the accuracy of ECM and other some diagnostic methods in detection of residual caries. In the result of present study, we found that the specificity of ECM was higher (0.73) than other two diagnostic methods but its sensitivity was the lowest. In the literature, the range of specificity value of ECM is from 0.56 to 0.98 [16, 43, 44]. The specificity value of ECM in present study is in accordance with the literature [16, 43, 44]. It has been reported that the range of sensitivity value is from 0.39 to 0.97 for dentinal caries [16]. In this study, the sensitivity value (0.47) of ECM locates in the literature range. ECM device can prevent the overexcavation with high specificity but its low sensitivity value can cause to remain carious lesions under the restoration. The use of ECM can be advised in the scanning examinations because of its higher specificity. Electrical measurements are showing early promise as a technique for the clinical detection of caries in clinical practice.

Previously, usefulness of DIAGNOdent for the detection of residual caries during excavation has been reported by Lussi et al. [27]. Also, Hossain et al. [45] recommended a combination of hardness testing by an explorer and DIAGNOdent for the assessment of carious dentin removal. The distribution of DIAGNOdent values in primary teeth exhibits a difference in an unimportant degree with respect to that of permanent teeth [46]. The best performance of DIAGNOdent is said to be in dentinal caries which is emerged by opening the fissure [31] and to predict the extension of caries lesions, mainly at dentinal caries threshold in primary teeth [21]. Gurbuz et al. [30] reported that DIAGNOdent and visual tactile examination can detect the remaining dentin as sound after bur excavation, but that DIAGNOdent abilities to detect residual caries were low after hand excavation and chemomechanical caries removal and, therefore, it is advisable to test the residual caries with an additional diagnostic tool such as visual tactile examination. Differently, in their study, DIAGNOdent readings have been used to observe the condition of the dentin after removal of the caries-infected dentin tissues that were stained with caries detecting dye. However, Lennon et al. [19] and Yazici et al. [20] reported that LF had greater sensitivities than CDD in assessment of residual caries. In this study, similar to previous studies, we found that the highest average sensitivity was shown in the measurements of LF (0.85) for detection of residual caries. In the study of Yazici et al. [20], the specificity value (0.86) was higher than sensitivity value (0.63) of LF. Contrarily, in the study of Lennon et al. [19], the sensitivity value (0.88) was higher than specificity value (0.70). Similar to the study of Lennon et al. [19], in our study, the sensitivity value (0.85) was higher than specificity value (0.50). In LF investigations on occlusal surface, some researchers reported that the sensitivity value was higher than specificity value [14, 33, 47, 48]. LF, which has been high sensitivity, is a valuable adjunct to clinical examination to detect the residual caries in the cavity after excavation. According to these results, LF can prevent to remain of carious lesions under the restoration. An ideal diagnostic method should offer, among other characteristics, high sensitivity and high specificity. However, these conditions are difficult to achieve with the available methods. Normally, a very high specificity is obtained at expense of reduced sensitivity. Likewise, an increase in sensitivity will be accompanied by decrease in specificity (increase in the false-positive diagnosis). Considering that rise in the false-positive proportion can be dangerous as it can lead to overtreatment, a technique that offers high specificity even at the expense of a slight reduction in sensitivity seems to be more appropriate [49].

In this present study, for LF, lower specificity values at D_3  _level caries when compared to sensitivity were found. Deep lesions contain more protein because of bacterial deposits and would therefore be more affected by denaturing. Thus, the increased fluorescence reflects the increased organic component in a lesion. Also, in this study, the teeth with deep dentin caries were used; higher readings may be therefore. This finding supports the proposal by Shi et al. [13] and Hibst and Paulus [50] that increased readings of LF reflects, the increased organic material in a deep lesion. The storage temperature and storage medium were shown to influence LF readings [50, 51]. Hibst and Paulus [50] reported that LF readings dramatically increased after storage of the teeth in 10% neutral-buffered formalin instead of saturated thymol saline. They suggested that formalin denatured the proteins, changing the structure and quality of one or more organic components, resulting in increased fluorescence [50]. Lussi et al. [51] demonstrated that the fluorescence values and hence the cut-off values decreased when the teeth were stored in thymol, chloramines, or formalin but remained stable when they were frozen during storage and were defrosted for fluorescence measurements [52]. In this study, the teeth were stored in saline solution without any antibacterial substance at 8°C. The cause of low specificity value of LF may be that teeth were kept in saline storage medium and at 8°C. The sensitivity values of LF were higher than other diagnostic tests and, therefore, it is advisable to detect residual caries in cavity, but the specificity values of LF were low; therefore, in addition to LF, residual dentin in deep dentin region must be evaluated as well as according to its hardness, color, and so forth.

The intraobserver reproducibility values of LF ranged from 0.75 to 0.94 for smooth and occlusal surfaces in the literature. In this study for LF, average intraobserver agreement was 0.50 and interobserver agreement was 0.48. Yazici et al. [20] found that intraobserver agreement was 0.93 and interobserver agreement was 0.61 for LF. The reproducibility of LF is lower than the literature range in this study. The lower reproducibility may be resulted from the experience with the use of LF device of observers. Therefore, the experience is an important factor in use of LF device and the measurements of LF have technical susceptibility to errors. Repeated measurements can be advised to prevent the variability in measurements. In this study, ECM had the highest intraobserver agreement value (0.86) but its interobserver agreement value was the lowest. The intraobserver agreement value was 1 and interobserver agreement value was 0.92 for CDD but the accuracy values of these devices were significantly lower than LF in this study. Furthermore, there was a significant difference between the measurements of ECM, CDD, and gold standard.

## 5. Conclusion

LF cannot detect the remaining dentin as sound (low specificity); therefore the use of LF could lead to over-preparation cavities. However, LF abilities to detect residual caries (sensitivity) were high and therefore it could be effective to evaluate the residual caries. For detection of residual caries, LF should be used with an additional diagnostic tool and it should be more improved to avoid excessive removal of the affected dentin.

## Figures and Tables

**Table 1:** Average sensitivity and specificity values (maximum and minimum) of CDD, LF, and ECM for detection of residual caries.

Groups		Observers		
		1	2	3
LF	Sensitivity	0.84 (0.80–0.87)	0.86 (0.82–0.89)	0.85 (0. 78–0.91)
	Specificity	0.60 (0.60–0.60)	0.40 (0.40–0.40)	0.50 (0.40–0.60)
	Accuracy	0.80 (0.78–0.82)	0.81 (0.78–0.84)	0.81 (0.76–0.86)
ECM	Sensitivity	0.30 (0.30–0.30)	0.49 (0.47–0.51)	0.62 (0.62–0.62)
	Specificity	0.70 (0.80–0.60)	0.90 (1.00–0.80)	0.60 (0.60–0.60)
	Accuracy	0.37 (0.38–0.36)	0.54 (0.52–0.56)	0.62 (0.62–0.62)
CDD	Sensitivity	0.56 (0.56–0.56)	0.51 (0.51–0.51)	0.58 (0.58–0.58)
	Specificity	0.60 (0.60–0.60)	0.60 (0.60–0.60)	0.60 (0.60–0.60)
	Accuracy	0.56 (0.56–0.56)	0.52 (0.52–0.52)	0.52 (0.52-0.52)

**Table 2:** Kappa values of inter- and intraobserver repeatability for CDD, LF, and ECM.

Observers	CDD	LF	ECM
1	1.00	0.58	0.86
2	1.00	0.50	0.80
3	1.00	0.43	0.91
1 versus 2	0.92	0.47	0.23
1 versus 3	0.96	0.56	0.34
2 versus 3	0.88	0.40	0.51
